# The phenotypic and functional study of tissue B cells in respiratory system provided important information for diseases and development of vaccines

**DOI:** 10.1111/jcmm.16278

**Published:** 2021-01-22

**Authors:** Li Fan, Qiongli Wu, Shuangpeng Kang, Binyan Yang, Changyou Wu

**Affiliations:** ^1^ Institute of Immunology Zhongshan School of Medicine Sun Yat‐sen University Guangzhou China; ^2^ Medical College Hubei University of Arts and Science Xiangyang China; ^3^ Clifford Hospital Jinan University Guangzhou China

**Keywords:** BCG, B_RM_, intranasal vaccination, respiratory system, tissue B cells

## Abstract

The field of tissue‐resident B cells has received increasing attention, yet the feature of tissue B cells in respiratory system is unclear. Here, we first show that non‐circulating B cells obtained from nasal, trachea and lung tissues are numerically and phenotypically distinct from their circulating counterparts. Analysis of single cell transcriptome sequence identified multiple differentially expressed genes between non‐circulating B cells and circulating B cells, which illustrated their heterogeneity. Furthermore, we found high expression of CXCR3 on non‐circulating B cells, and the chemokine CXCL11 was also up‐regulated in the respiratory tissues, suggesting that CXCR3‐CXCL11 axis might accelerate the local resident of non‐circulating B cells in respiratory tract. Interestingly, intranasal immunization with BCG in mice elicited a sustained humoral immune response via induction of IgA and IgG Abs, which revealed the role of B cells. Meanwhile, tissue‐resident B cells, IgA^+^ and IgG^+^ memory B cells (MBCs) in respiratory tissues, as well as plasma cells in bone marrow, were expanded and maintained, and these subsets probably developed into antibody‐producing cells to participate in the local humoral immunity. Our data illustrate the phenotype and function of tissue B cells in the upper and lower airways, provide references for the prospective development of vaccines.

## 1 INTRODUCTION

In recent years, tissue‐resident memory T cells (T_RM_) have been clarified, which put tissue B cells or tissue‐resident memory B cells (B_RM_) onto the topic. In fact, the lack of unique markers on MBCs in mice limits further extensive research.^1^, ^2^ The respiratory system is the first line that contacts with inhalant allergens, and some diseases spread through the respiratory tract and seriously affect people's health, such as influenza and asthma.^3^, ^4^ Numerous studies have demonstrated that T_RM_ in nasal and lung tissues perform faster and stronger cellular immune in situ than do circulating T cells.^5^, ^6^, ^7^ However, few studies are focused on tissue B cells in respiratory tract.

Early studies had suggested that lung flu‐specific B cells and MBCs were characterized by high expression of CD69.^8^ More recent studies report that B_RM_ cells induced in the lungs are phenotypically and functionally distinct from their counterpart circulation_,_ such as high expression of CXCR3, complete lack of CD62L, quick respond and production of Abs after secondary influenza infections.^9^ Like that of T_RM_ cells, B_RM_ cells are also necessary to prevent respiratory viruses or infections. These findings guarantee the dominant role of tissue B cells or B_RM_ cells at the local sites. Therefore, better understanding of the diversities between tissue B cells in respiratory tract and their systemic counterparts provides a basis for the treatment of more respiratory diseases.

Tuberculosis (TB) caused by the intracellular pathogen *Mycobacterium tuberculosis* (*M tb*) is still a major health threat worldwide.^10^, ^11^ BCG, the only licensed TB vaccine, executes limited protection against infection.^12^ Despite several animal and clinical cases including our laboratory have demonstrated that specific T cells are acquired to pulmonary TB on account of mycobacteria to survive within the phagocytes,^13^, ^14^ the potential roles of B cells and Abs in TB are largely undefined. Many researchers believed that BCG vaccination induced Ab responses had no effective protection against TB. Fortunately, progressive study found an increase of specific IgG levels from South African infants after BCG vaccination, which reduced risk of *M tb* infection.^15^ In a DBA/2 mouse model, the targeting delivery through intranasal BCG challenge generates superior protection against TB and increases the levels of specific and non‐specific IgA in lungs.^16^ Intranasal vaccination of mice with BCG also produces significantly higher levels of *M tb*‐specific IgA, IgG1 and IgG2a Abs in the nasal lavage fluid (NLF) compared to subcutaneous immunization of mice and declines the bacteria loaded in the lungs.^17^ Others showed that compared to the subcutaneous delivery system, the route of mucosal immunization induced not only local mucosal but also systemic immune responses.^18^ In consideration of its effect on the location and differentiation of immunization‐primed cells, intranasal vaccination is deemed as a viable strategy to facilitate mucosal immune response against some respiratory diseases. Although previous studies are concentrated on the local lung tissues, it is worth noting the contribution of B cells and Abs in the field of respiratory system.

In this study, we aimed to conduct the immunophenotype and function of tissue B cells in respiratory system. Our results suggested that the numbers, phenotypes and gene expression of non‐circulating B cells in nasal, trachea and lung tissues were distinct from circulating B cells in blood. The possible mechanism of non‐circulating B cells that colonized the respiratory tissues was mediated by CXCR3‐CXCL11 axis. We further investigated that intranasal‐vaccined mouse model with BCG induced the production of non‐specific and antigen‐specific IgA and IgG Abs in the airway, and tissue‐resident B cell subsets, MBCs and plasma cells were also increased and maintained. In summary, these data hint that tissue B cells are involved in regulating the local immune response of respiratory system and Abs production to enhance protective immunity via mucosal transport system.

## 2 MATERIALS AND METHODS

### 2.1 Mice

Female C57BL/6 mice were obtained from the Laboratory Animal Center of Sun Yat‐sen University (SYXK 2015‐0107) and were housed in a specific pathogen‐free condition. Mice used were six‐ to eight‐week‐old and killed under deep isoflurane (RWD life science, China) anaesthesia. Animal experiments were approved by the Experimental Animal Ethics Committee of Sun Yat‐sen University, Guangzhou, China.

### 2.2 Antigen and immunization

In brief, 50 μg BCG/mouse (purchased from the Institute of Biological Products, Chengdu, China) suspended in PBS was dripped into the nasal cavity of mice that were anaesthetized with isoflurane. The immunization process was shown in Figure 6A.

### 2.3 Intravascular staining in vivo

To distinguish non‐circulating B cells and circulating B cells, the fluorochrome‐conjugated mAb was used to label circulating cells in vivo as previously reported.^19^, ^20^ Briefly, 3 μg of anti‐CD45 Ab (clone 30‐F11, BD Bioscience) diluted in 200 μL of PBS was intravenously injected into mice for 5 min prior to euthanasia. Isolated cells from nasal, trachea and lung tissues for staining, which labelled with anti‐CD45 Ab, were considered as in the circulation, and the counterpart was non‐circulating cells for further study.

### 2.4 Tissue preparation and cell isolation

Peripheral blood mononuclear cells and splenocytes were isolated by Ficoll‐hypaque (Tianjin Hao Yang Biological Manufacture, Tianjin, China) density gradient centrifugation at 216*g* for 20 minutes at room temperature. Cells from bone marrow were treated with red blood cell lysis buffer. Nasopharyngeal‐associated lymphoid tissues (NALT) from soft palate were mechanically mashed through 70 μm cell strainers.

Nasal (which was isolated from the skull of mice, including nasal cavity and nasal turbinates, and cutted out the excess tissues and bones of nasal passages), trachea and lung tissues were dispersed in cold PBS, gently triturated with multifunction filter (MagicFilter, Bozhen Technology, China). Subsequently, cell suspension was passed through 40 μm cell strainers and further isolated by Percoll (GE Healthcare, Sweden) density gradient centrifugation at 280*g* for 20 minutes. These mononuclear cells were collected and then suspended in completed RPMI 1640 medium.

### 2.5 Cell culture

To explore the change of surface markers on B cells, sorted CD19^+^IgD^+^CD62L^+^, CD19^+^IgD^+^CD23^+^ and CD19^+^IgM^+^IgD^+^B cells from the splenocytes were marked by CFSE and were cultured for 4 days or 7 days with LPS (0.5 µg/mL, Sigma‐Aldrich) and anti‐CD40 (1 µg/mL, BD Biosciences) in the presence of IL‐2 (20 ng/mL, R&D systems) at 37◦C with 5% CO_2_.^21^ The expression of CD62L, CD23, IgD or IgM was analysed.

### 2.6 Flow cytometry and mAbs

To analyse the cellular composition in different tissues, cell staining was performed for 30 min at 4℃ in the dark with fluorescent mAbs as described previously.^22^ Before staining, cells were washed with staining buffer containing 0.1% BSA and 0.05% sodium azide and blocked with CD16/32 Ab for 15 min on ice to reduce non‐specific binding. For surface molecular detection, the following mAbs were used (all from BD Biosciences, Thermo Fisher Scientific and Biolegend): CD45‐FITC/PE (30‐F11), CD19‐PerCp‐Cy5.5/PE‐Cy7 (1D3), CD3‐PE‐CF594 (145‐2C11); CD103‐PE/PE‐Cy7 (2E7), CD69‐PE/PE‐Cy7 (H1.2F3), CD62L‐APC (MEL‐14), CD138‐PE (281‐2), IgM‐APC‐Cy7 (II/41), IgM‐PECF594 (R6‐60.2), IgD‐APC (11‐26c, 2a), IgG‐FITC (Poly4060), IgG‐PE‐Cy7 (Poly4053), IgA‐PE (mA‐6E1), IgA‐FITC (C10‐3 ), CD23‐PE (B3B4), CXCR3‐PE (CXCR3‐173), CXCR5‐PE‐Cy7 (2G8), CX3CR1‐PE (SA011F11), CCR5‐PE (HM‐CCR5), CD80‐PE (16‐10A1), CD24a‐FITC (M1/69), CD38‐FITC/PerCp‐Cy5.5 (90). Dead cells were excluded by Helix NP™NIR (Biolegend) staining. Cell samples were performed on FACS Aria II (BD Biosciences), and data were analysed by FlowJo10 (TreeStar, San Carlos, CA, USA).

### 2.7 Sample collection

The serum was prepared following a standard protocol.^23^ The supernatants of bronchoalveolar lavage fluids (BALF) were obtained from the lungs of mice by trachea intubation rinsing with 900 μL of PBS. The nasal lavage fluids (NLF) were collected by washing nasal cavity twice with 125 μL of sterile PBS.^24^ These samples were stored at −80℃ until detection.

### 2.8 ELISA

Collected serum, BALF and NLF were measured by ELISA for the detection of total IgG and IgA levels following the manufacturer's protocols (Invitrogen, USA). Levels of antigen‐specific IgA and IgG in serum, BALF and NLF were also assayed using ELISA kits. The 96‐well plates were pre‐coated with 10 μg/mL BCG that mixed in PBS overnight at 4℃. The following procedure referred to the detection of total IgG and IgA levels. The results were shown with OD values.

### 2.9 Quantitative real‐time PCR

For comparison of the gene expression, total RNA from nasal, trachea, lung and blood were extracted by TRIzol (Invitrogen) and subsequently reverse‐transcribed using a cDNA Synthesis Supermix kit (novoprotein scientific Inc). The mRNA levels of CXCL9, CXCL10, CXCL11 and CXCL13 were evaluated by SYBR Green probes (novoprotein scientific Inc) in the Step One Plus^TM^ Real‐Time PCR System. The real‐time PCR cycle steps were 95°C for 1 minute, 95°C for 20 seconds and 60°C for 1 minute, 40 cycles. The following primer sequences were used: CXCL9 Forward: 5′‐CATCATCTTCCTGGAGCAGTGTGG‐3′, Reverse: 5′‐AGTCTTCCTTGAACGA CGACGAC‐3′; CXCL10 Forward: 5′‐AATCATCCCTGCGAGCCTATCC‐3′, Reverse: 5′‐TGTGCGTGGCTTCACTCCAGTT‐3′; CXCL11 Forward: 5′‐GAACAGGAAGG TCACAGCCATAGC‐3′, Reverse: 5′‐GAGGCGAGCTTGCTTGGATCTG‐3′; CXCL13 Forward: 5′‐GGCCACGGTATTCTGGAAGC‐3′, Reverse: 5′‐ACCGACAA CAGTTGAAATCACTC‐3′; GAPDH Forward: 5′‐ATGACCACAG TCCATG CCAT CAC‐3′, Reverse: 5′‐ATGCCTGCTTCACCACCTTCTTG‐3′. The 2^‐ΔΔCT^ method was used for result calculation.

### 2.10 Analysis of single cell transcriptome sequence

Differentially expressed mRNA of single cell at different state can be found using variance analysis strategy. The samples from lung tissues and blood were obtained from six mice, and non‐circulating B cells and circulating B cells were prepared by flow cytometry staining and sorting. The total numbers of non‐circulating B cells and circulating B cells were both less than or equal to 1000. Then, the cells were washed, and lysis buffer containing ribonuclease inhibitor was added to amplify by the Smart‐Seq2 method. Purified amplification product was further constructed the library. Qualified libraries were loaded on Illumina Hiseq platform.

The analysis process of Annoroda single‐cell transcriptome sequence information was mainly divided into three parts: quality control of sequencing data, data comparison analysis and transcriptome deep analysis. The amount of each gene was calculated by Fragments per Kilobase per Million Mapped Fragments (FPKM = 103*F/NL/10^6^). Differentially expressed genes were performed with DEGseq package. The multiple testing adjusted P‐value (FDR 5%) was to assess whether these genes were significantly differential expressed.

### 2.11 Bioinformatics analysis

Using R package for hierarchical cluster analysis of the differentially expressed genes was shown in the heat map. The network analysis of gene‐biological process term in Gene Ontology (GO) database was built, and our analysis showed the top 20 of biological process terms and constructed gene‐biological process network by the Cytoscape software.

### 2.12 Statistical analysis

Statistical graphs were made with GraphPad Prism 8 (GraphPad Software Inc). The unpaired Student's t test was used to compare two groups, and one‐way ANOVA including Sidak's multiple comparisons test and Dunnett's multiple comparisons test was for more than two groups. Data were shown as minimum and maximum values or mean ± SD. Significant P‐values were exhibited in figures: ****P < .0001; ***P < .001; **P < .01; *P < .05.

## 3 RESULTS

### 3.1 Identification and distribution of non‐circulating B cells in the respiratory tissues

**FIGURE 1:**
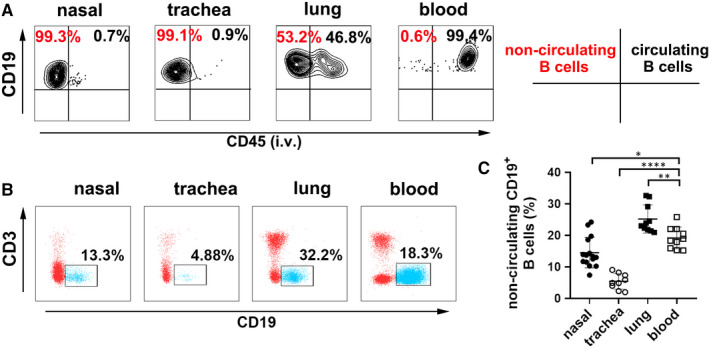
Identification and distribution of non‐circulating B cells in the respiratory tissues. (A) CD45^‐^CD19^+^B cells (non‐circulating B cells) and CD45^+^CD19^+^B cells (circulating B cells) from nasal, trachea and lung tissues were identified by tail vein injection with fluorochrome‐conjugated CD45 antibody in naive mice. (B) Live, singlet CD45^‐^ lymphocytes from nasal, trachea and lung tissues and CD45^+^ lymphocytes from blood were gated and subsequently analysed on CD19^+^B cells by flow cytometry. (C) The frequencies of non‐circulating B cells in nasal, tracheal, lung tissues and circulating B cells in blood were shown as individual data points as well as mean ± SD and compared with Dunnett's multiple comparisons test for multiple comparisons. *P < 0.05, **P < 0.01; ****P < 0.0001

To characterize non‐circulating B cells in nasal, trachea and lung tissues, we evaluated cellular labelling of fluorochrome‐conjugated anti‐CD45 Ab by flow cytometry. The results showed 99.4% of CD45^+^CD19^+^B cells in blood, suggesting that circulating cells in vivo were successfully marked. More than 99% of B cells from nasal and trachea tissues were CD45^‐^CD19^+^ (non‐circulating B cells), non‐circulating and circulating B cells in lung tissues were almost equal distribution (Figure 1A). Meanwhile, we observed that the frequencies of non‐circulating B cells in nasal (14.506 ± 4.84%) and trachea (5.4 ± 2.47%) tissues were significantly lower than that of circulating B cells in blood (19.18 ± 3.43%), whereas in lung tissues were much higher (25.66 ± 4.47%, *P* < .01) (Figure 1B,C).

### 3.2 Phenotypic difference of non‐circulating B cells in the respiratory tissues and circulating B cells in blood

**FIGURE 2:**
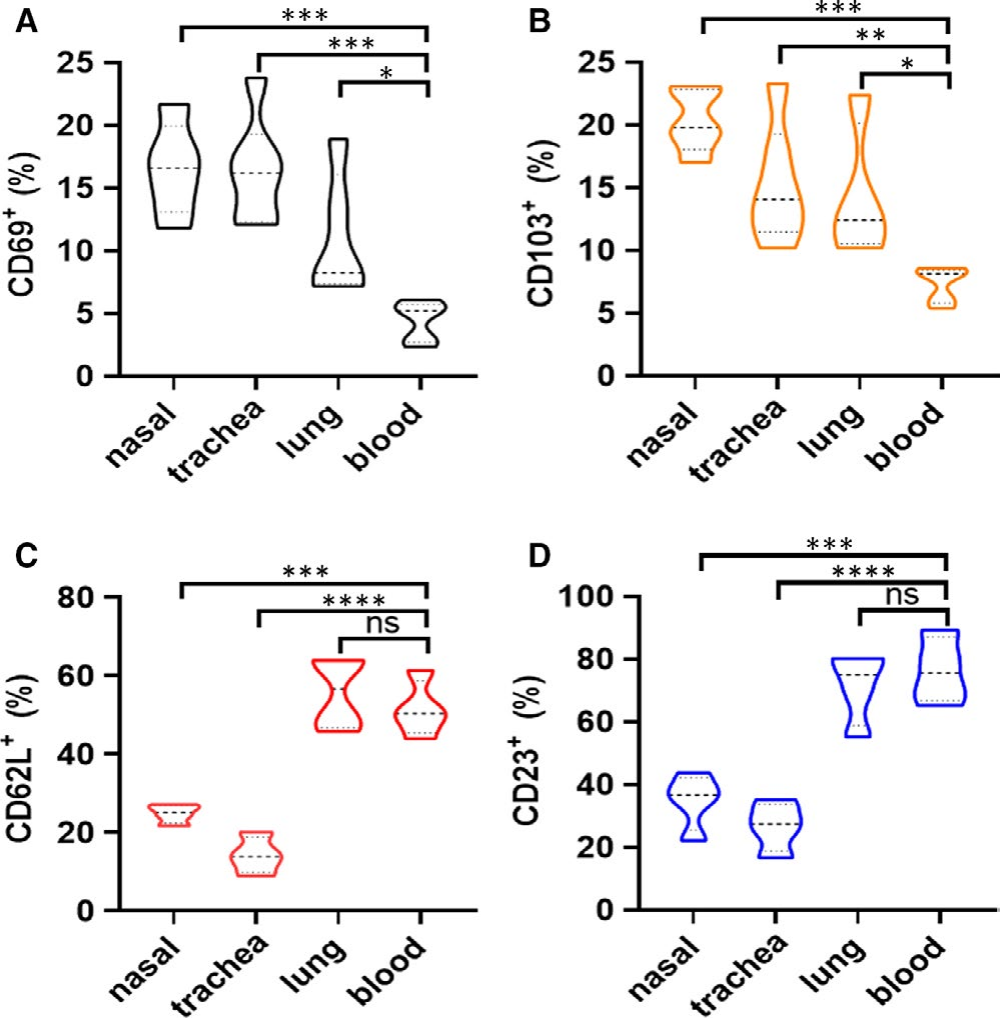
Phenotypic difference of non‐circulating B cells in nasal, trachea, lung tissues and circulating B cells in blood. (A‐D) Gated on live and singlet cells, the expression of CD69, CD103, CD62L and CD23 on non‐circulating CD19^+^ B cells in nasal, trachea, lung tissues and circulating B cells in blood was analysed by flow cytometry. The panels showed their proportions at these sites, respectively. All data were representative of more than four independent experiments by six mice each group. Significance was compared with Dunnett's multiple comparisons test for multiple comparisons. *P < 0.05, **P < 0.01; ***P < 0.001; ****P < 0.0001; ns, no significance

To further explore whether phenotype of non‐circulating B cells in nasal, trachea, lung tissues and circulating B cells in blood was different, we compared the expression levels of typical markers such as CD69, CD103, CD62L and CD23. Notably, the expression of CD69 and CD103 was up‐regulated on non‐circulating B cells in three tissues compared with that of circulating B cells in blood (Figure 2A, B). In contrast, frequencies of CD62L and CD23 were reduced on non‐circulating B cells in three tissues except lungs compared with that of circulating B cells in blood (Figure 2C, D). Based on the phenomenon, we extended the experiment to analyse the change of CD62L and CD23 on B cells. Data shown in Figure S1 suggested that the expression of CD62L and CD23 was decreased after B cells activation. These results indicate that phenotypes of non‐circulating B cells in tissues are different from circulating B cells in blood, and implying that non‐circulating B cells in these tissues are kept in a more activated state.

### 3.3 Expression of surface immunoglobulins (Igs) on non‐circulating B cells in three tissues and circulating B cells in blood

**FIGURE 3:**
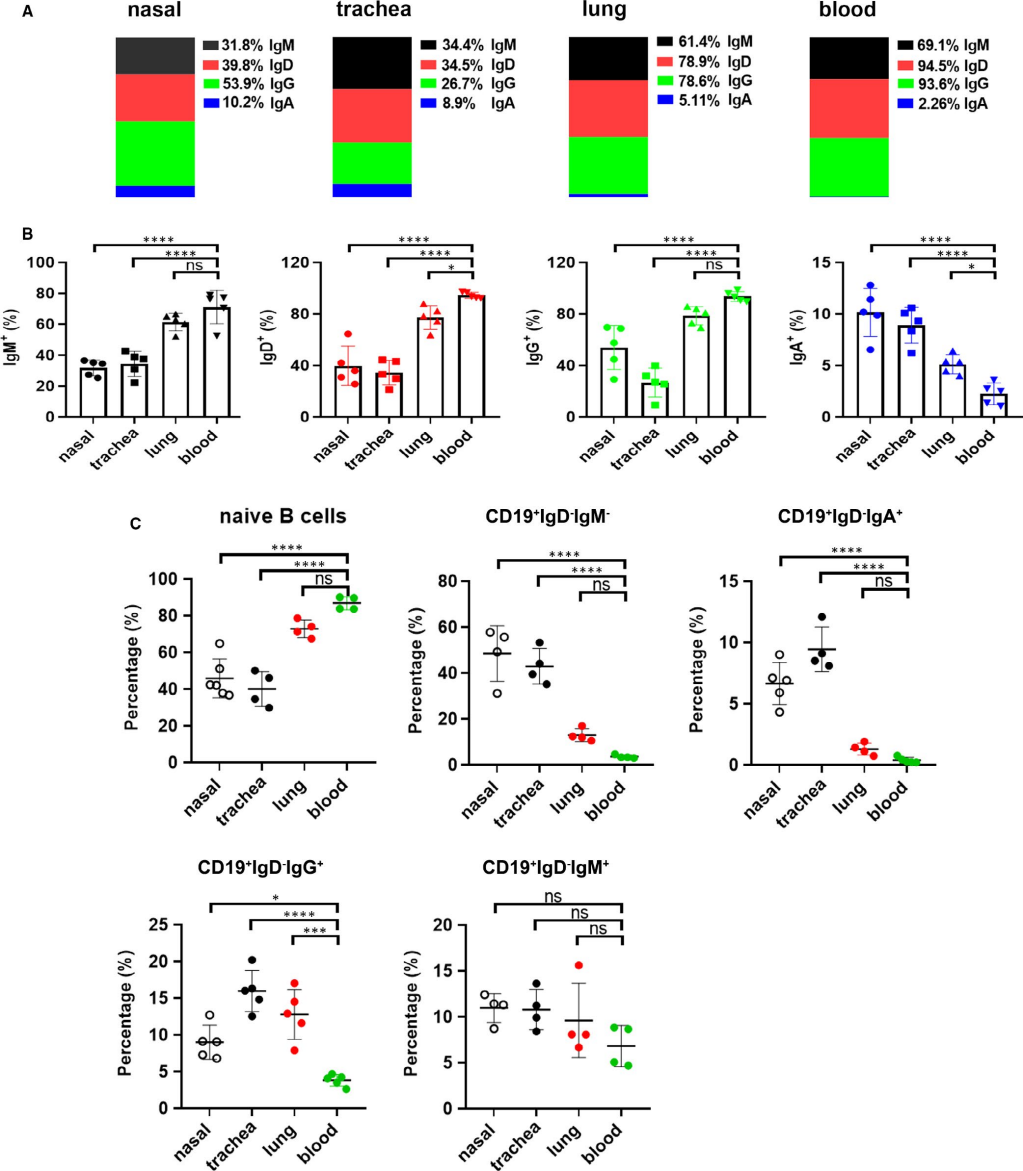
Expression difference of surface Igs on non‐circulating B cells from nasal, trachea, lung tissues and circulating B cells from blood. (A) The representative diagram showed the means of IgM (black), IgD (red), IgG (green) and IgA (blue) expression on live and singlet non‐circulating B cells in nasal, trachea, lung tissues and circulating B cells in blood. (B) Statistical results represented five independent experiments by seven mice each group. (C) The cells from nasal, trachea and lung tissues were gated on live and singlet non‐circulating lymphocytes, and the frequencies of naïve B cells, CD19^+^IgD^‐^IgM^‐^, CD19^+^IgD^‐^IgA^+^, CD19^+^IgD^‐^IgG^+^ and CD19^+^IgD^‐^IgM^+^ cells were analysed by flow cytometry, respectively. Statistical charts from four independent experiments by seven mice each group were shown. Data were compared with Dunnett's multiple comparisons test of one‐way ANOVA. *P < 0.05, ***P < 0.001; ****P < 0.0001; ns, no significance

After investigation on phenotype, we ulteriorly elucidated the expression of surface Igs. Non‐circulating B cells in nasal and trachea tissues except lungs had a dramatical decrease of IgM and IgG expression relative to circulating B cells in blood. Likewise, the amount of IgD on non‐circulating B cells in three tissues also showed a marked reduction. Although surface IgA expression on non‐circulating B cells was relatively low, which was higher in three tissues than that in blood (Figure 3A, B). The reason may be that the airway belongs to the mucosal site where is rich in IgA.

Next, we pinpointed the distribution of naïve B cells and memory B cells at these sites. The results showed that the proportions of naive B cells (CD19^+^IgD^+^) in nasal and trachea tissues except lungs were significantly lower than those of blood, and frequencies of CD19^+^IgD^‐^IgM^‐^ (isotype switched B cells) and CD19^+^IgD^‐^IgA^+^ (IgA^+^ MBCs) were significantly higher than those in blood. Meanwhile, the proportions of CD19^+^IgD^‐^IgG^+^ (IgG^+^ MBCs) in three tissues were significantly higher than those in blood. The distribution of CD19^+^IgD^‐^IgM^+^ (IgM^+^ MBCs) was not significantly different between three tissues and blood (Figure 3C).

### 3.4 The gene expression profile in non‐circulating and circulating B cells

**FIGURE 4:**
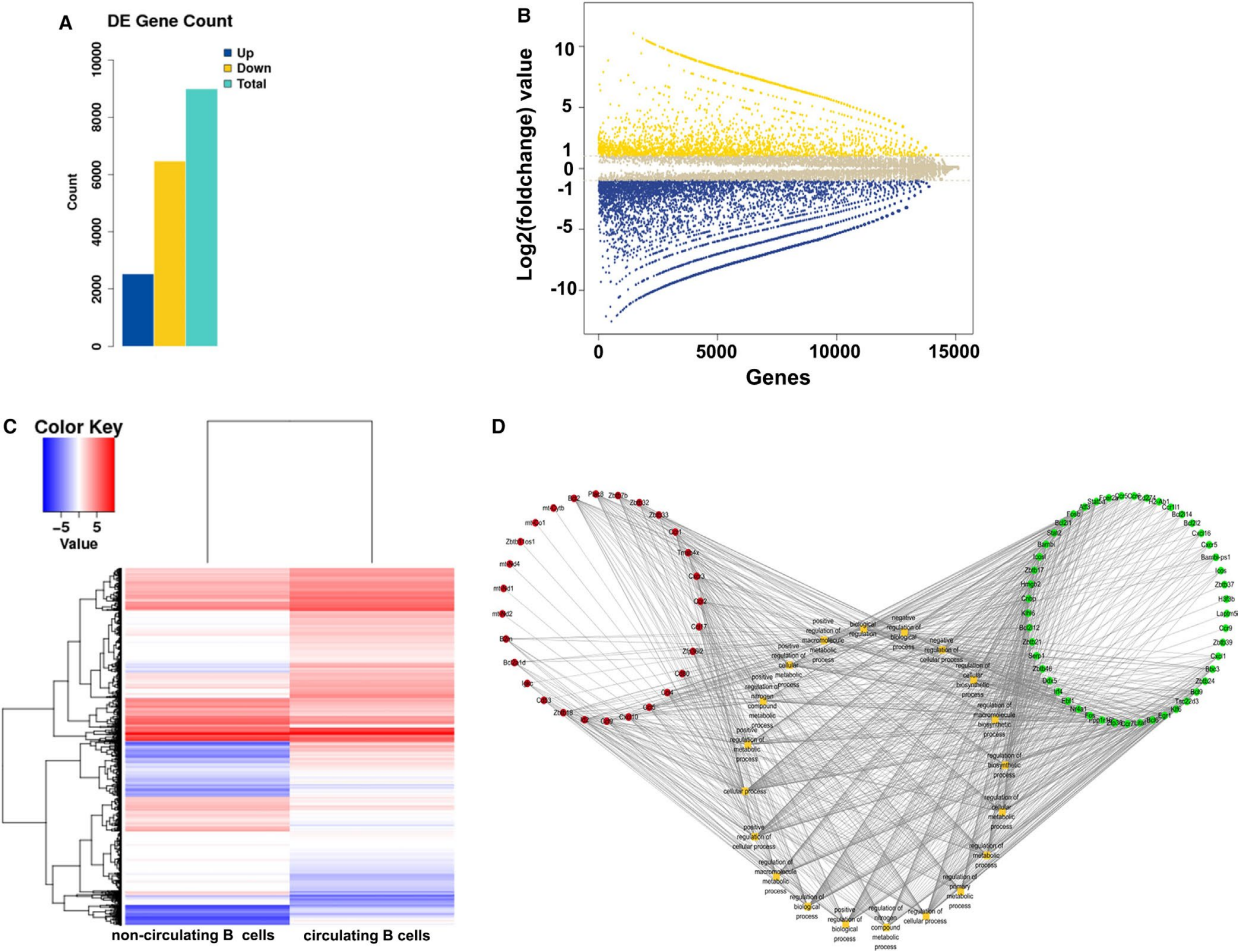
Identification of differentially expressed genes between non‐circulating B cells and circulating B cells. (A) The statistical data of up‐regulated and down‐regulated genes between non‐circulating B cells and circulating B cells. (B) Volcano plot of differentially expressed genes between non‐circulating B cells and circulating B cells. The longitudinal line represents FC value. Yellow and blue symbols indicate down‐regulation and up‐regulation, respectively. (C) The Hierarchical cluster heat map of differentially expressed genes between non‐circulating B cells and circulating B cells. Red displays relative high expression, and blue indicates low expression. (D) The coexpression network of differentially expressed genes and the top 20 of GO terms. A round node represents a gene. Yellow rectangle node indicates a biological process term, red indicates up‐regulated genes and green down‐regulation

Based on the above study that numbers and phenotypes of non‐circulating B cells in tissues were distinct from circulating B cells, signifying that non‐circulating B cells and circulating B cells might exert different functions. Therefore, we intended to clarify their heterogeneity. Single cell transcriptome sequnence was used for the analysis of their gene expression. Our samples were identified 2525 up‐regulated and 6467 down‐regulated genes, in non‐circulating B cells versus circulating B cells based upon the standard of FC ≥ 2 for up‐regulation and ≤ 0.5 for down‐regulation, as well as *P* < .05 (Figure 4A, B). In addition, the heat map depicted all differentially expressed genes (Figure 4C).

Coexpression networks can annotate the function of unknown genes and analyse the genome‐wide of complex functional structure in biological system. We selected partial genes with the most remarkable changes and combinated with our study, a total of 77 differentially expressed genes. These genes involved in the potential biological processes were further illustrated by bioinformatics analysis. As shown in Figure 4D, the coexpression network was consisted of 28 up‐regulated genes, 49 down‐regulated genes and the top 20 enriched biological process terms. The analysis showed that one gene participated in multiple biological processes, such as CXCR3 and its ligand CXCL10.

### 3.5 High expression of CXCR3 on non‐circulating B cells in respiratory tract respond to CXCL11

**FIGURE 5:**
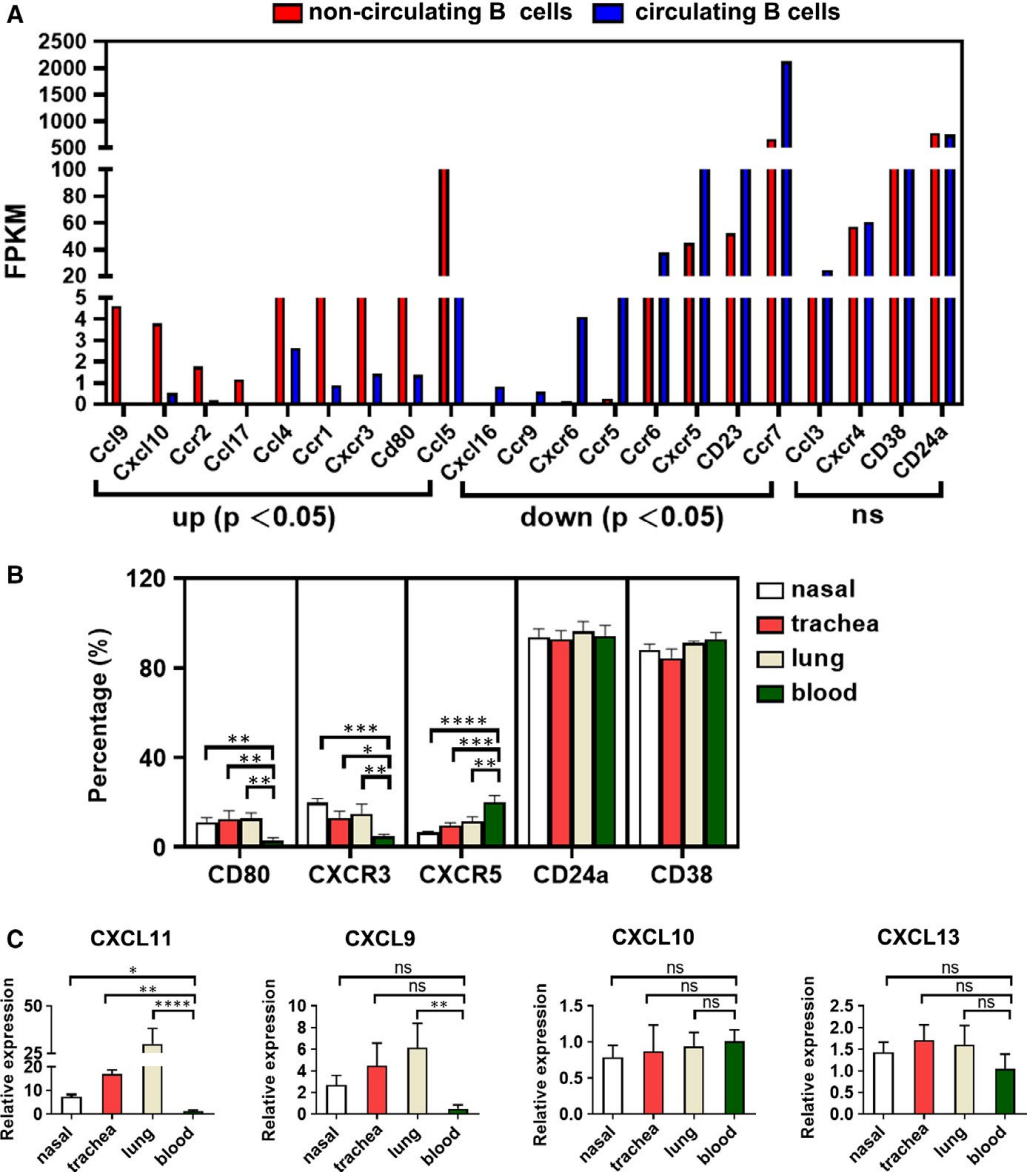
High expression of CXCR3 on non‐circulating B cells in the respiratory tissues respond to CXCL11. (A) The expression amount of chemokine receptors, chemokines and surface molecules in non‐circulating B cells (total numbers ≤ 1000) and circulating B cells (total numbers ≤ 1000) were shown. ‘up’ means up‐regulated genes, ‘down’ means down‐regulated genes, and ‘ns’ means no significant changes. *P* < 0.05 represents statistical significance. (B) The frequencies of CD80, CXCR3, CXCR5, CD24a and CD38 on non‐circulating B cells in nasal, trachea, lung tissues and circulating B cells in blood were detected by flow cytometry. Data were obtained from more than three independent experiments for seven mice each group. (C) The mRNA levels of chemokine CXCL9, CXCL10, CXCL11 and CXCL13 in nasal, trachea, lung tissues and blood were quantified by RT‐PCR. Data were representative of more than four independent experiments for four mice each group. Statistical significance was shown as mean ± SD and compared with Dunnett's multiple comparisons test for multiple comparisons. *P < 0.05, **P < 0.01; ***P < 0.001; ****P < 0.0001; ns, no significance

The analysis of single‐cell transcriptome sequnence revealed that various chemokine receptors, chemokines and surface molecules were differentially expressed between non‐circulating B cells and circulating B cells. Expression of partial genes were shown in Figure 5A, including 9 up‐regulated genes, 8 down‐regulated genes and 4 genes with no significant differences. The vertical axis represented their expression amount (FPKM) in each group.

In order to verify the expression of these genes, we further analysed the frequencies of CD80, CXCR3, CXCR5, CD24a and CD38 on non‐circulating B cells from nasal, trachea, lung tissues and circulating B cells from blood by flow cytometry. As we expected, compared to circulating B cells from blood, the expression of CD80 and CXCR3 was significantly increased, CXCR5 was decreased, whereas CD24a and CD38 was comparable on non‐circulating B cells from three tissues (Figure 5B). Together, our results were consistent with single cell transcriptome sequnence.

To further establish the possible mechanism of non‐circulating B cells retained in the respiratory tissues, we compared the expression difference of various chemokine receptors. Based on the results in Figure 5B, we observed that the percentage positivity of CXCR3 was higher and CXCR5 was lower on non‐circulating B cells in respiratory tract than that of circulating B cells in blood. In addition, the expression levels of CX3CR1 and CCR5 were not significantly different between them (Figure S2). Hence, we suspected that the resident of non‐circulating B cells in the airway was related to CXCR3. To test this conjecture, the mRNA levels of chemokines CXCL9, CXCL10, CXCL11 (CXCR3) and CXCL13 (CXCR5) in nasal, trachea, lung tissues and blood were examined by RT‐PCR. Interestingly, only the CXCL11 levels in three tissues were prominently higher than those in blood (Figure 5C). Overall, these results support the possibility that the resident of non‐circulating B cells in respiratory tract may be via CXCR3/CXCL11 axis.

### 3.6 Intranasal immunization with BCG induces a long‐term specific humoral immune response

**FIGURE 6:**
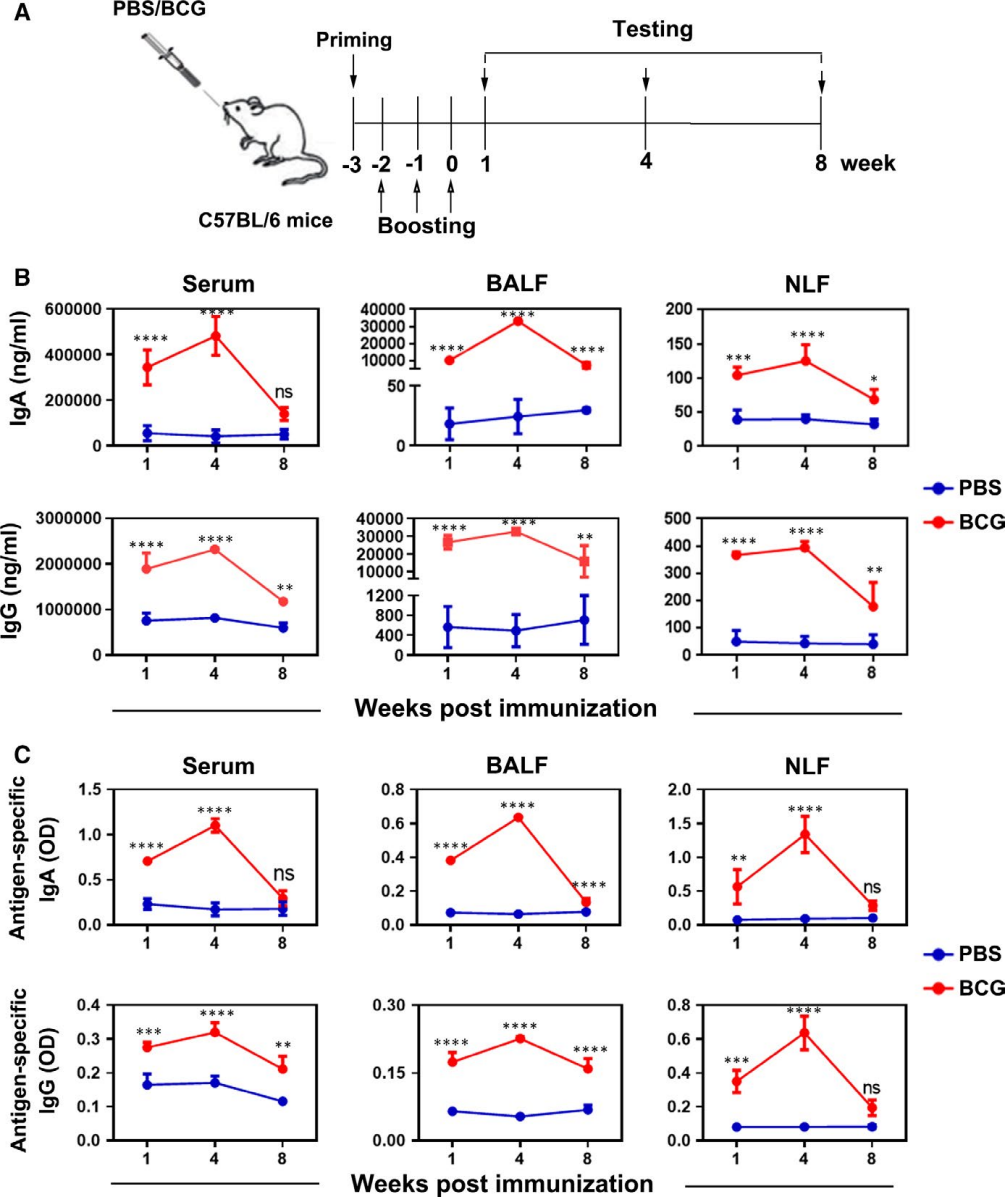
Intranasal vaccination with BCG induced long‐term humoral immune response. Mice were challenged intranasally with BCG or treated with PBS as a control once a week for 4 weeks. The samples of serum, BALF and NLF from BCG‐vaccinated group and PBS group were harvested at the indicated times. (A) The schedule of intranasal vaccination with BCG in mice and detection timeline was shown, n = 7‐10/group. (B) The levels of IgA and IgG Abs in serum, BALF and NLF were detected by ELISA. (C) The 96‐well plates were pre‐coated with 10 μg/mL BCG in PBS, the levels of antigen‐specific IgA and IgG Abs from serum, BALF and NLF were examined by ELISA. Data were shown as the OD values. Statistical results were representative of at least three independent experiments by seven mice each group and analysed by Sidak's multiple comparisons test. *P < 0.05; **P < 0.01; ***P < 0.001; ****P < 0.0001; ns, no significance

To understand the roles of B cells in the respiratory tissues, we immunized mice intranasally with BCG following the protocol, and PBS was used as a control group (Figure 6A). At the indicated times, total Ig levels in the serum, BALF and NLF were measured by ELISA. Data noted that the total levels of secreted IgA and IgG in the serum, BALF and NLF from mice immunized with BCG were higher than those from PBS group. The production of Abs peaked at week 4 after last boosting and maintained for 8 weeks, except for IgA production in serum, which decreased earlier (Figure 6B). Meaningfully, we found that these IgA and IgG from serum, BALF and NLF were also antigen‐specific Abs. However, only the antigen‐specific IgA and IgG production in BALF was both kept for at least 8 weeks (Figure 6C). Our data indicate that intranasal immunization with BCG form a sustained humoral immunity at the mucosa sites of the airway.

### 3.7 Intranasal immunization with BCG preferably expands non‐circulating MBCs in respiratory tract and plasma cells in bone marrow

**FIGURE 7:**
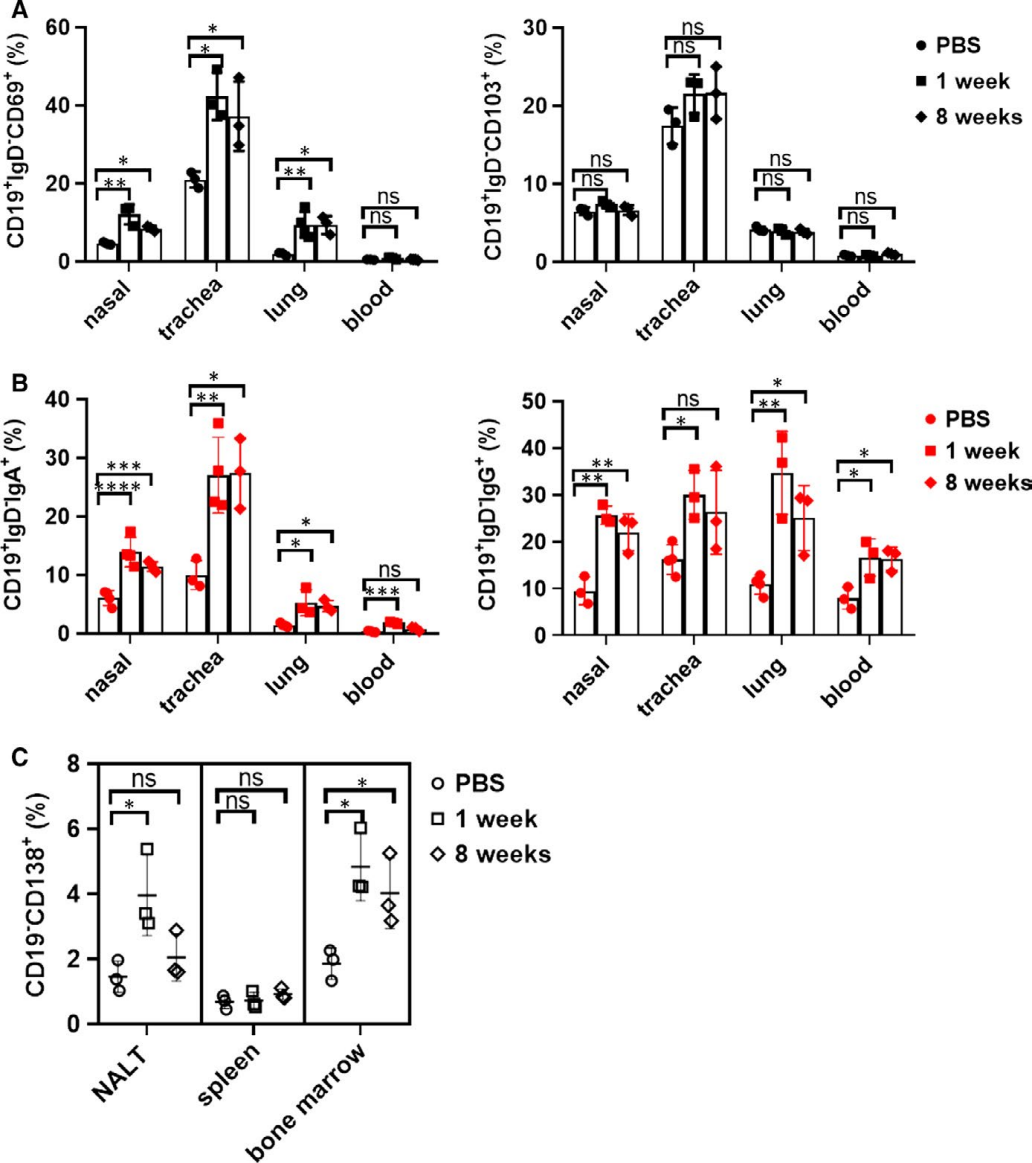
Intranasal immunization expanded the frequencies of non‐circulating MBCs in respiratory tract and plasma cells in bone marrow. Mice were intranasally vaccinated with BCG and killed at week 1 and 8 after last boosting. (A) Gated on live and singlet CD45^‐^CD19^+^ B cells from nasal, trachea and lung tissues or CD45^+^CD19^+^ B cells from blood in PBS or BCG group, and then analysed the percentages of CD19^+^IgD^‐^CD69^+^ and CD19^+^IgD^‐^CD103^+^ cells by flow cytometry, the graphs from at least three independent experiments displayed their percentages in each group. (B) The frequencies of non‐circulating CD19^+^IgD^‐^IgA^+^ and CD19^+^IgD^‐^IgG^+^MBCs from three tissues were assessed by flow cytometry. (C) Cells from NALT, spleen and bone marrow were analysed by flow cytometry for the distribution of CD19^‐^CD138^+^ plasma cells at different time‐points. Data were representative of at least three independent experiments, and statistical significance was compared with Dunnett's multiple comparisons test for multiple comparisons. *P < 0.05; **P < 0.01; ***P < 0.001; ****P < 0.0001; ns, no significance

As non‐circulating B cells rather than circulating B cells had a increased expression of CD69 and CD103, we speculated that there was a subset of tissue‐resident B cells exerting immune response in respiratory tract. Indeed, the respiratory tissues showed higher frequencies of CD19^+^IgD^‐^CD69^+^ cells and CD19^+^IgD^‐^CD103^+^ cells than did blood. However, in mice immunized with BCG, only percentages of CD19^+^IgD^‐^ CD69^+^ cells were increased and maintained for 8 weeks in the nasal, trachea and lung tissues (Figure 7A), which suggested the local importance of CD19^+^IgD^‐^CD69^+^ cells in the respiratory tract.

However, consistently with the quantitative Ab assay, the numbers of non‐circulating CD19^+^IgD^‐^IgA^+^ and CD19^+^IgD^‐^IgG^+^ MBCs from nasal, trachea and lung tissues were also expanded at week 1 after last boosting and kept for 8 weeks (Figure 7B). Those IgA^+^ and IgG^+^ MBCs might differentiate into Ab‐secreting cells until congenic antigen exposure to secret antibodies against infection.

The majority of long‐lived plasma cells in lymphoid organs and bone marrow could produce effective antibodies for systemic protection. Therefore, we further compared the frequencies of plasma cells after BCG immunization. Except for spleen, CD19^‐^CD138^+^ plasma cells were both increased in NALT and bone marrow at week 1 after last boosting and remained for at least 8 weeks in bone marrow (Figure 7C). These observations suggest that plasma cells induced by intranasal immunization with BCG may survive in the bone marrow for a long time to bring the immune protection.

## 4 DISCUSSION

Paralleling to T_RM_ cells, increasing evidence have manifested that tissue B cells or B_RM_ cells from lungs are involved in the immune response of influenza protection. ^8^, ^9^ B_RM_ cells in lungs are phenotypically distinct from MBCs in lymphoid tissues.^9^ Consistently with these studies, we found that non‐circulating B cells from the respiratory tissues had a difference in the phenotype and gene expression compared with circulating B cells from blood. Furthermore, intranasal immunization with BCG could elicited a long‐term humoral immune response, highlighting the action of tissue B cells in respiratory system.

More than 99% of B cells in the nasal and trachea tissues were non‐circulating B cells; in contrast, only the half were non‐circulating B cells in lungs owing to the vasculature. The percentages of non‐circulating B cells in the respiratory tissues were different from circulating B cells in blood. CD69 and CD103, the markers of tissue‐resident T cells, are lowly or not expressed in circulating T cells.^25^, ^26^, ^27^ We found that the percentage positivity of CD69 and CD103 on non‐circulating B cells in three tissues rather than circulating B cells in blood had a significant increase, and many studies reported that lung flu‐specific B cells, MBCs and B_RM_ also highly expressed CD69.^8^, ^9^ However, non‐circulating B cells in the expression of CD62L and CD23 were lower than those from circulating B cells in blood. Results were consistent with the in vitro assays that the expression of CD62L and CD23 was decreased with the activation of B cells. Actually, effector memory T cells and B_RM_ cells also lack CD62L. CD23, the low affinity IgE Fc receptor, is expressed on all mature B cells and involved in B cell activation.^28^, ^29^ These results indicate that tissue non‐circulating B cells highly express tissue‐resident markers and keep in a more activated state, which help a faster and stronger local humoral immune response when expose to infection.

The synthesis of surface Ig promotes the differentiation of B cells into plasma cells. IgM, IgD, IgG and IgA on non‐circulating B cells in the respiratory tissues and circulating B cells in blood were differentially expressed. In contrast to blood, the increase of IgA^+^ non‐circulating B cells and IgA^+^ MBCs showed the superiority of IgA Ab at the mucosal sites, whereas the reduction of IgD^+^ non‐circulating B cells in three tissues might be the consequence of their activation. Traditionally, it had been accepted that surface IgD was decreased or lost after B cells differentiated into MBCs. Indeed, we observed that in vitro surface IgD but not IgM on B cells was decreased with their activation and division, suggesting that IgD could be considered as an important marker of B cell activation (Figure S3). These findings support that tissue non‐circulating B cells are sustained in a more active state and show the distributional difference of naive B cells and MBCs between respiratory tissue and blood.

Simultaneously, elucidating the heterogeneity between non‐circulating B cells and circulating B cells contribute to study the function. To date, single cell transcriptome sequence has become an indispensable tool in the field of immunology.^30^, ^31^ We used this method for the identification of differentially expressed genes and further predicted the biological processes by GO analysis, discovering that one gene involved in multiple biological processes. These unique genes may mediate different effector functions, the local persistence and survival of non‐circulating B cells.

Given our observation that there were significant differences in the gene expression of various chemokine receptors, chemokines and surface molecules on non‐circulating B cells and circulating B cells, which agreed with flow cytometry analysis. Moreover, non‐circulating B cells in the tissues exhibited the up‐regulation in CXCR3 expression, which is a chemokine receptor that interacts with CXCL9, CXCL10 or CXCL11.^32^ Meaningfully, CXCR3 was highly expressed on both flu‐specific MBCs and B_RM_ from lung tissues.^8^, ^9^ Although our data showed that only the CXCL11 levels were significantly enhanced in three tissues compared with that in blood, supported a potential mechanism that the resident of non‐circulating B cells in the respiratory tissues might be mediated by CXCR3‐CXCL11 axis. Interaction between chemokines and chemokine receptors may facilitate the synergy of immune cells at these tissue sites.

What roles might B cells play in the respiratory tract? A series of studies have documented that IgM, IgG1, IgG3 and IgA are involved in the protection of *M tb* infection.^33^, ^34^, ^35^, ^36^, ^37^, ^38^, ^39^ Meanwhile, the route of intranasal BCG vaccination exceeded subcutaneous route and preferably elicited non‐specific and specific IgA Abs in the lungs of TB mice.^16^ Chen et al ^40^ also showed that the immune response induced by intranasal BCG vaccination might be necessary in lungs. To enrich our knowledge, a mouse model was established to expound that the intranasal BCG vaccination imparted humoral immunity in respiratory system. We observed the increase of non‐specific and specific IgA and IgG Abs in the airway that was consistent with others.^16^, ^17^ However, these Abs could not be sustained in NLF, one possibility is that their maintainment required continuous antigen stimulation.

In animal models, mucosal BCG vaccination shaped lung T_RM_ cells exerting faster and stronger protection against TB.^41^, ^42^ We also found the existence of tissue‐resident CD19^+^IgD^‐^CD69^+^ and CD19^+^IgD^‐^CD103^+^ B cells, and the numbers were much higher in respiratory tract than those in blood, but only the increase of CD19^+^IgD^‐^CD69^+^ cells induced by intranasal BCG vaccination was maintained for at least 8 weeks in three tissues. Our previous study illustrated that B cells in the pleural fluid of TB patients could respond to *M tb*‐specific antigen and impinge the local immune response.^43^ These data manifested the role of B cells in the local TB infection, even so, further studies are still needed to dissect the protective mechanism of CD19^+^IgD^‐^CD69^+^ cells. It is possible that a minute number of BCG‐specific CD19^+^IgD^‐^CD69^+^ cells in the respiratory tissues are sufficient to transfer against *M tb* infection, which will be similar to the roles of influenza virus‐specific CD8^+^ T cells in respiratory tract.^44^ Therefore, efforts to induce the local formation of tissue‐resident B cells or B_RM_ in the development and design of TB vaccine may contribute to improving vaccine effectiveness.

Moreover, the predominant increase and retention of non‐circulating IgA^+^ and IgG^+^MBCs was observed in the respiratory tissues. Even though these MBCs appeared to be associated with antigen‐specific IgA and IgG production, we had no direct evidence. Long‐lived plasma cells in the lymph nodes and bone marrow secreted efficient Abs to protect the system. Plasma cells also increased and remained in bone marrow for up to 8 weeks after intranasal immunization with BCG, suggesting that the expanded plasma cells could survive in bone marrow to execute long‐lasting immune protection. Unfortunately, we cannot prove the origin of plasma cells and whether they are sustained for life.

In this study, we provide important information concerning phenotype, gene expression and functional capacity of non‐circulating B cells from the respiratory tissues and circulating B cells from blood. However, this study still requires to consolidate by extending the detection times and evaluating the protective effect of intranasal vaccination on *M tb* infection. Nonetheless, our preliminary studies not only enrich the immunological theory of B cells in respiratory system but also help the development of a new vaccine.

## CONFLICTS OF INTEREST

Authors guaranteed no conflicts of interest.

## AUTHOR CONTRIBUTION

**Changyou Wu:** Writing‐review & editing (equal). **Li Fan:** Resources (equal); Software (equal); Writing‐original draft (equal); Writing‐review & editing (equal). **Qiongli Wu:** Software (equal). **Shuangpeng Kang:** Software (equal). **Binyan Yang:** Software (equal).

## AUTHORSHIP

Changyou Wu and Li Fan: Research design. Li Fan: Research and data analysis. Qiongli Wu, Shuangpeng Kang and Binyan Yang: Flow cytometry analysis and scientific planning. Li Fan and Changyou Wu: Writing and revising. All authors: Approval of the final manuscript.

## Supporting information

Fig S1‐S3
