# Supplementary material for: The phenotypic and functional study of tissue B cells in respiratory system provided important information for diseases and development of vaccines
Source: J Cell Mol Med. 2021 Jan 22;25(5):2621–32. doi: 10.1111/jcmm.16278 (PMC7933955; doi:10.1111/jcmm.16278)
Supplement: Supplementary file 1 — Fig S1‐S3 [file JCMM-25-2621-s001.docx]

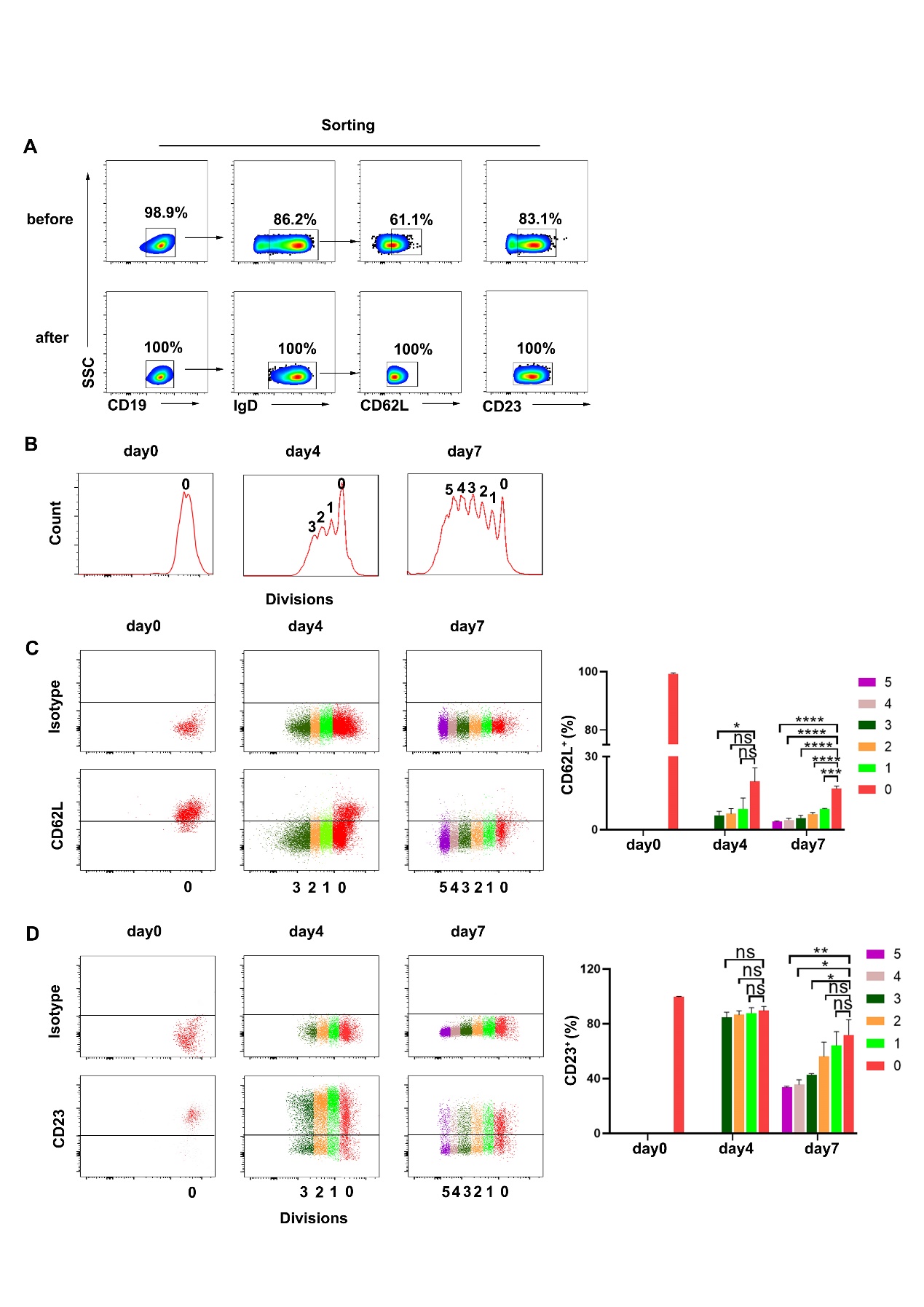


**Supplementary Figure S1.** The expression of CD62L and CD23 on B cells during activation and division. (A) Isolated splenocytes were stained, CD19^+^IgD^+^CD62L^+^ and CD19^+^IgD^+^ CD23^+^ cells were further sorted by flow cytometry, their purity was up to 100%. (B) Purified CD19^+^IgD^+^CD62L^+^ and CD19^+^IgD^+^CD23^+^ B cells were marked by CFSE and stimulated with LPS, CD40 plus IL-2 for 4 days and 7 days. Cells were collected at the indicated time and detected their divisions by flow cytometry. (C & D) The expression of CD62L and CD23 on CD19^+^IgD^+^CD62L^+^ and CD19^+^IgD^+^CD23^+^ B cells with the divisions was analyzed by FlowJo10. Statistical data from three independent expriments were shown and compared with one-way ANOVA for multiple comparisons. **P* < 0.05, ***P* < 0.01; ****P* < 0.001; *****P* < 0.0001; ns, no significance.


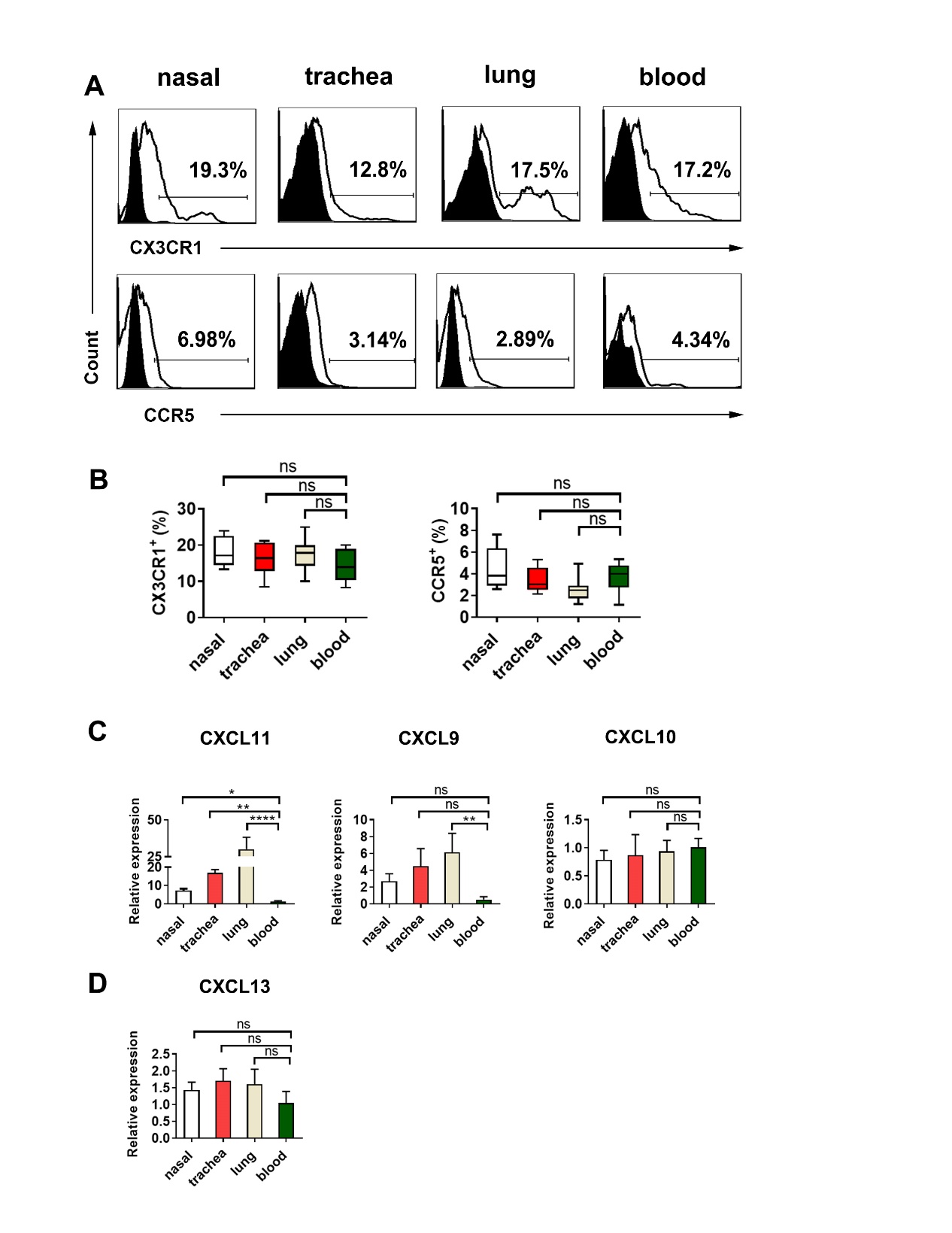


**Supplementary Figure S2.** The expression of chemokine receptors CX3CR1 and CCR5 in the respiratory tissues. (A & B) Representative hisgram showed the expression of chemokine receptors CX3CR1 and CCR5 on non-circulating B cells in nasal, trachea, lung tissues and circulating B cells in blood, respectively. Statistical results of their percentages were shown. Data were representative of more than four independent experiments for seven mice each group. Statistical significance was shown as minimum and maximum values, compared with one-way ANOVA. ns, no significance.


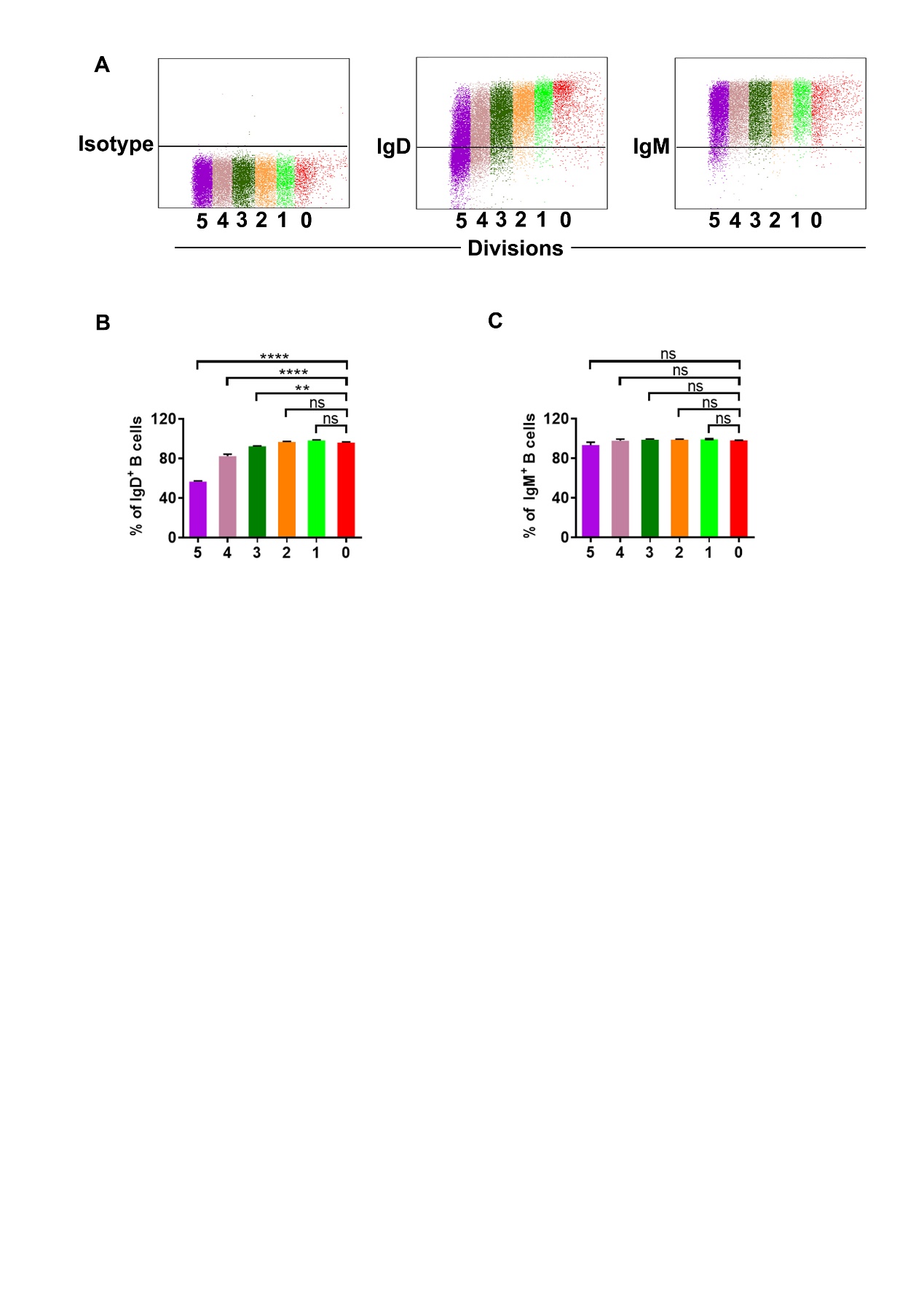


**Supplementary Figure S3.** The expression of IgD and IgM on B cells after activation.

(A) CD19^+^IgM^+^ IgD^+^ cells were sorted by flow cytometry and marked with CFSE, and further stimulated with LPS, CD40 in the presence of IL-2 for 7 days. Cells were collected for detecting the expression of IgD and IgM by flow cytometry. Isotype was as a negative control. Statistical results showed the frequencies of IgD^+^ and IgM^+^ on CD19^+^IgD^+^IgM^+^ cells in every division. (B & C) Data showed three independent expriments and statistical significance was analyzed by one-way ANOVA for multiple comparisons. ***P* < 0.01; *****P* < 0.0001; ns, no significance.
